# Capsaicin Receptor TRPV1 Delays Aortic Aging in Atherosclerotic Mice by Inhibiting the ISG15‐p53 Pathway

**DOI:** 10.1111/jcmm.71157

**Published:** 2026-04-25

**Authors:** Duo Zhang, Jishuang Liu, Te Liu, Jiulin Chen, ZhiHua Yu, Yun Gu, Fangfang Dou

**Affiliations:** ^1^ Shanghai Geriatric Institute of Chinese Medicine Shanghai University of Traditional Chinese Medicine Shanghai China; ^2^ Longhua Hospital Shanghai University of Traditional Chinese Medicine Shanghai China

**Keywords:** atherosclerosis model mice, ISG15, TRPV1, vascular aging, vascular endothelial cells

## Abstract

Vascular aging constitutes a significant pathological basis for the aging of various organs and systems within the human body. This study employed a murine model of atherosclerosis (AS) and Transient Receptor Potential Vanilloid 1 (TRPV1)‐deficient AS mice to ascertain whether TRPV1 exerts an influence on AS by modulating aortic aging. Furthermore, the study sought to expand upon the molecular mechanisms by which TRPV1 regulates aortic aging. TRPV1 expression was examined in aortic tissue of *Apoe* ^−/−^ mice, and its expression level was significantly reduced in comparison with that of control mice. *Apoe* ^−/−^ *Trpv1* ^−/−^ mice were subjected to a high‐fat diet, after which a significant increase in aortic sinus senescent cell area, senescence‐associated secretory phenotypes (SASP) and expression of senescence‐associated proteins was observed, accompanied by an increase in plaque area and instability. Transcriptome sequencing ascertained that the deletion of TRPV1 led to a substantial augmentation in the expression of ubiquitin‐like protein 15 (ISG15) within the aortic tissue. It then demonstrated that TRPV1 deletion promotes ISG15‐induced aortic senescence using *Apoe* ^−/−^ *Trpv1* ^−/−^ high‐fat dietary mice, as well as cellular experiments. Studies utilising PPI analysis and in vitro cultured EA.hy926 cells have demonstrated that ISG15 promotes vascular endothelial cell senescence by facilitating the expression and phosphorylation of p53 and p21, and by impeding the phosphorylation of retinoblastoma (Rb). Aortic TRPV1 deficiency promotes aortic senescence by promoting upregulation of ISG15 expression, which in turn promotes p53 and p21 phosphorylation and inhibits Rb phosphorylation.

## 1 Introduction

Vascular aging constitutes the pathological basis of a considerable number of age‐related diseases, with a strong correlation to the development of cardiovascular diseases [1]. As people get older, arteries can undergo structural and functional changes, including increased stiffness, remodelling of the arterial walls, loss of angiogenic capacity, and endothelium‐dependent vasodilatory dysfunction. These changes can increase a patient's risk of cardiovascular events and mortality [2]. At present, a significant number of studies are concentrating on investigating the mechanisms of vascular aging and its association with a variety of diseases. It is evident that functional alterations in endothelial cells play a pivotal role in the development of cardiovascular disease and the process of vascular aging [3]. Consequently, the study of vascular aging is imperative to comprehending its association with cardiovascular disease and to identifying methods to delay or reverse it.

Transient Receptor Potential Vanilloid 1 (TRPV1) is a non‐selective cation channel that is widely present in mammalian sensory nerve endings and in a variety of tissues [4]. Since their initial discovery, the functions of TRPV1 channels in both physiological and pathological processes have been the focus of significant research interest. It is important to note that this system is not only responsive to conventional stimuli such as temperature, pain, and inflammation, but is also implicated in the onset and progression of a wide range of diseases, including cardiovascular, neurodegenerative, and digestive diseases [5, 6, 7, 8]. Cardiovascular diseases (CVDs) are a major cause of morbidity and mortality on a global scale. In recent years, there has been an increasing focus on TRPV1 in the study of cardiovascular diseases, with the receptor being implicated in a variety of physiological and pathological processes, including the regulation of vascular tone, the promotion of angiogenesis, and the modulation of antifibrotic, anti‐inflammatory, and antioxidant responses [9]. In microglia, capsaicin has been demonstrated to alter mitochondrial Ca^2+^ concentration and its membrane potential by acting on the TRPV1 receptor located on the mitochondrial membrane. Changes in the concentration of Ca^2+^ have been observed to stimulate mitochondria, resulting in the production of reactive oxygen species (ROS). These ROS have been shown to induce mitochondrial migration by acting on the mitogen‐activated protein kinase (MAPK) signalling pathway [10, 11]. In cardiomyocytes, capsaicin, akin to cyclosporin A (a calmodulin phosphatase inhibitor), has been observed to reduce mitochondrial membrane potential and diminish infarct size in myocardial infarction model animals by modulating the dephosphorylation/phosphorylation equilibrium of TRPV1 channels [12, 13]. In the *Apoe* ^−/−^ high‐fat diet model of AS, capsaicin has been observed to impede mitochondrial dysfunction in the aortic vasculature, thus attenuating cardiac damage [14]. The extant literature suggests that the activation of TRPV1 receptors has a beneficial effect on the maintenance of mitochondrial function, thereby producing cardiovascular protection.

ISG15 is a ubiquitin‐like protein (UBL) encoded by the interferon (IFN)‐stimulated gene 15. In recent years, significant progress has been made in the functional studies of ISG15, revealing its important role in cellular homeostasis, immune response, and cancer [15]. It has been hypothesised that ISG15 fulfils a multifaceted role in cellular processes, functioning not only as a free intracellular or extracellular molecule but also contributing to the regulation of protein translation, autophagy, exosome secretion, cytokine secretion, cytoskeletal dynamics, the DNA damage response, telomere shortening, and immunomodulation [16]. Nevertheless, the potential mechanisms and molecular consequences of ISG15 and ISG15 covalent modification (ISGylation) remain to be fully elucidated. ISGylation of p53 at multiple sites has been found to promote tumour growth by degrading p53‐targeted proteasomes [17]. ISG15 gene deletion has been demonstrated to result in the accumulation of misfolded and dominant‐negative p53, leading to the inhibition of overall p53 activity. This, in turn, has been shown to result in a reduction in DNA damage‐induced senescence, accelerated cell proliferation, and reduced p21 expression [18]. The majority of extant research on ISG15 has focused on antiviral immunity [19], antibacterial infection [20], and modulation of tumourigenesis [21, 22]. The role of ISG15 in other diseases through modulation of cellular homeostasis and the immune response has received comparatively less attention. Consequently, an in‐depth understanding of the role of ISG15 in the pathogenesis of various human diseases is imperative for the development of effective treatments and interventions.

The present study reports that the inhibition of TRPV1 channels promoted aortic aging and exacerbated atherosclerosis progression in atherosclerosis model mice by promoting ISG15 expression, elevating phosphorylation of p53 and p21, and inhibiting phosphorylation of Rb.

## 2 Materials and Methods

### 2.1 Experimental Animals and Cells

The study utilised a total of 20 male C57BL/6J mice, aged 8 weeks, with a body mass range of 20–25 g, and 20 male *Apoe* ^−/−^ mice, aged 8 weeks, with a body mass range of 20–25 g. ApoE−/− mice exhibit a spontaneous tendency towards atherosclerosis; therefore, some experiments have been conducted with C57BL/6J mice as controls [23]. Existing literature indicates that the employment of male mice in atherosclerosis models is considered a standard procedure [24, 25]. These mice were procured from Beijing Charles River Laboratory Animal Technology Co. The Animal Production Certificate No. SCXK (Jing) 2016–0006 was granted. The animals were housed in the SPF grade animal laboratory of Shanghai University of Traditional Chinese Medicine (SUTCM) under the following conditions: a 12 h/12 h alternating light and dark environment, temperature (24 ± 1) °C, relative humidity 50%–70%, and free access to food and water during the experimental period. The experimental operation procedures strictly adhered to the relevant regulations and provisions of the Ethics Committee for Animal Experiments of SUTCM, and the experiment successfully passed the ethical review of animal experiments of SUTCM, with the ethical number PZSHUTCM220124017. Eighteen male *Apoe* ^*−/−*^ *Trpv1* ^*−/−*^ mice, aged 8 weeks and with a body mass of 20–25 g, were provided by Researcher Zhihua Yu, Department of Pharmacology and Chemical Biology, School of Medicine, Shanghai Jiao Tong University. C57BL/6 mice were utilised as controls, with no intervention being implemented. The experimental model group consisted of *Apoe* ^−/−^ mice that were fed a high‐fat diet (Shanghai Fanbo Biotechnology Co. Ltd., Batch No.: D12492) for a period of 3 months. *Apoe* ^*−/−*^ *Trpv1* ^*−/−*^ mice were similarly fed a high‐fat diet for a period of 3 months. *Apoe* ^*−/−*^ *Trpv1* ^*−/−*^ ISG15i mice were administered an ISG15 AAV9 interference virus (Shanghai GenePharma) via tail vein injection for a period of 1 month, after which they were fed a high‐fat diet for a further 3 months. All mice were anaesthetised to facilitate heart and blood vessel extraction for experimental procedures.

The human umbilical vein cell line, EA.hy926, and the rat thoracic aortic smooth muscle cell line, A7r5, were procured from the Chinese Academy of Sciences Cell Bank (Shanghai). Cells were cultivated in vitro using DMEM (Dulbecco's Modified Eagle Medium) with 10% foetal bovine serum (FBS) (Gibco, USA). The inoculation of cells was conducted in a variety of vessel sizes, including 96‐well plates, 24‐well plates, 6‐well plates, and 10 cm Petri dishes, in accordance with the specific experimental requirements. The ISG15 overexpressing (oe‐ISG15) and interfering (i‐ISG15/ISG15i) AAV9 viruses, as well as the p53‐interfering AAV9 viruses, were procured from Shanghai GenePharma. The virus was introduced into in vitro‐cultured EA.hy926 cells with the aim of observing the infection efficiency under a fluorescence microscope. This observation was made after 48 h of infection. In addition, the expression levels of ISG15 and p53 proteins were observed by means of a western blot assay. This assay was used to clarify the expression and interference efficiency. The TRPV1 agonist capsaicin and the antagonist capsazepine were procured from MCE. Capsaicin was added at a final concentration of 1 μg/mL [26] and capsazepine was added at a final concentration of 5 μg/mL [27, 28] in in vitro cultured EA.hy926 cells or A7r5 cells. Oxidised low‐density lipoprotein (ox‐LDL) was added to EA.hy926 cells or A7r5 cells cultured in vitro as a cell injury model group at a concentration of 200 μg/mL. The experimental approach entailed the treatment of cultured cells with ox‐LDL for a duration of 24 h, a process that induced cellular lipid damage. This was followed by the administration of capsazepine or capsaicin to ascertain their effects on the lipid damage.

### 2.2 Transcriptome Sequencing

Following the administration of isoflurane (Shenzhen RWD Life Sciences Co. Ltd.) anaesthesia to mice, a systemic perfusion with 20 mL of physiological saline was performed. The heart and aorta were removed intact, with the surrounding adipose and connective tissue being trimmed away. The aorta is then to be minced meticulously using ophthalmic scissors. The tissue was placed in Trizol reagent (Invitrogen, USA) for rapid homogenisation; the resulting homogenate will then be used for RNA extraction. The RNA concentration and purity were detected by Nanodrop spectrophotometer. The process of sequencing the transcript was carried out by Shanghai Personalbio Biotechnology Co. Subsequent to the sequencing results. The process of sequencing the transcriptomes was conducted using a method of bulk sequencing, employing the MiSeq sequencer on the Illumina platform. The study utilised the DESeq software for the purpose of differential gene expression analysis, whereby the screening of differentially expressed genes was conducted in accordance with the following criteria: The log2FoldChange value was found to be greater than 1, and the *p*‐value was found to be < 0.05, thus meeting the established significance threshold. Differentially expressed genes and associated regulatory pathways were identified among the groups. The target genes were then verified by Real‐time PCR for the purpose of subsequent functional assays.

### 2.3 Real‐Time PCR

Total RNA was extracted from aortic tissues by grinding them in liquid nitrogen, adding 1 mL TRIzol Reagent (Thermo Fisher Scientific, USA) and homogenising them. The reverse transcription process was initiated using a reverse transcription kit (Servicebio, Wuhan), which facilitated the conversion of RNA into cDNA. The subsequent amplification of the target gene was conducted utilising a SYBR Green qPCR kit (Servicebio, Wuhan), adhering to the stipulated conditions. These conditions encompassed a pre‐denaturation step at 95°C for a duration of 5 min, denaturation at 95°C for 5 s, an annealing process at 55°C for 30 s, and an extension step at 72°C for 15 s for 40 cycles. GAPDH was selected as an internal reference gene. The mRNA content of the target genes was calculated by means of the 2^−ΔΔCt^ relative quantification method. All primer sequences were synthesised by Shanghai Sangon Biotechnology Company Limited, and these are shown in Table 1.

**TABLE 1:** Primers for real‐time PCR in *Mus musculus* (m), *Homo sapiens* (h) and *Rattus norvegicus* (r).

Gene	Forward primer	Reverse primer
*Trpv1*(m)	TGGCTCATATTTGCCTTCAG	CAGCCCTAGGAGTTGATGGA
*Il‐1β*(m)	GAAATGCCACCTTTTGACAGTG	TGGATGCTCTCATCAGGACAG
*Il‐6*(m)	CTGCAAGAGACTTCCATCCAG	AGTGGTATAGACAGGTCTGTTGG
*Tnf‐α*(m)	CTGAACTTCGGGGTGATCGG	GGCTTGTCACTCGAATTTTGAGA
*Pai‐1*(m)	TCTGGGAAAGGGTTCACTTTACC	GACACGCCATAGGGAGAGAAG
*Mmp‐3*(m)	TTAAAGACAGGCACTTTTGGCG	CCCTCGTATAGCCCAGAACT
*Ccl5*(m)	GCTGCTTTGCCTACCTCTCC	TCGAGTGACAAACACGACTGC
*Cxcl10*(m)	CCAAGTGCTGCCGTCATTTTC	TCCCTATGGCCCTCATTCTCA
*Mcp‐1*(m)	TAAAAACCTGGATCGGAACCAAA	GCATTAGCTTCAGATTTACGGGT
*Dkk1*(m)	CAGTGCCACCTTGAACTCAGT	CCGCCCTCATAGAGAACTCC
*Isg15*(m)	GGTGTCCGTGACTAACTCCAT	TGGAAAGGGTAAGACCGTCCT
*Tnfsf18*(m)	GTCATGGCTCTTGTGCATAGT	GATGGCAGTTGGCTTGAGTGA
*Gfra1*(m)	CACTCCTGGATTTGCTGATGT	AGTGTGCGGTACTTGGTGC
*Tgfb2*(m)	CTTCGACGTGACAGACGCT	GCAGGGGCAGTGTAAACTTATT
*Fbxo32*(m)	ACACATCCTTATGCACACTGG	TCTCCATCCGATACACCCACA
*Rsad2*(m)	TGCTGGCTGAGAATAGCATTAGG	GCTGAGTGCTGTTCCCATCT
*Sult1b1*(m)	GCACACCAGGTGACATTGTAA	CCGAGGTGATGGAGTTTTCTTC
*Cmpk2*(m)	ACTTGACCTAGTTGACCAGTGC	GCATCCAGTCCTTCAATGGC
*Oasl*(m)	TTGTGCGGAGGATCAGGTACT	TGATGGTGTCGCAGTCTTTGA
*Krt7*(m)	AGGAGATCAACCGACGCAC	GTCTCGTGAAGGGTCTTGAGG
*Efemp1*(m)	GCAATGCACCGATGGATATGA	TTTGGGCTGTTTTAGGAAGGC
*Ccn2*(m)	GACCCAACTATGATGCGAGCC	CCCATCCCACAGGTCTTAGAAC
*Apln*(m)	ACCAGGAGCCTTTTGTAGCC	CCTGACTCCCCCTACCCTAC
*Vcan*(m)	ACTAACCCATGCACTACATCAAG	ACTTTTCCAGACAGAGAGCCTT
*Fabp4*(m)	AAGGTGAAGAGCATCATAACCCT	TCACGCCTTTCATAACACATTCC
*Bgn*(m)	TGCCATGTGTCCTTTCGGTT	CAGGTCTAGCAGTGTGGTGTC
*Vcam1*(m)	AGTTGGGGATTCGGTTGTTCT	CCCCTCATTCCTTACCACCC
*Txnip*(m)	TCTTTTGAGGTGGTCTTCAACG	GCTTTGACTCGGGTAACTTCACA
*Top2a*(m)	CAACTGGAACATATACTGCTCCG	GGGTCCCTTTGTTTGTTATCAGC
*Gapdh*(m)	AGGTCGGTGTGAACGGATTTG	TGTAGACCATGTAGTTGAGGTCA
*Il‐1β*(h)	ATGATGGCTTATTACAGTGGCAA	GTCGGAGATTCGTAGCTGGA
*Il‐6*(h)	ACTCACCTCTTCAGAACGAATTG	CCATCTTTGGAAGGTTCAGGTTG
*Tnf‐α*(h)	CCTCTCTCTAATCAGCCCTCTG	GAGGACCTGGGAGTAGATGAG
*Pai‐1*(h)	ACCGCAACGTGGTTTTCTCA	TTGAATCCCATAGCTGCTTGAAT
*Mmp‐3*(h)	CGGTTCCGCCTGTCTCAAG	CGCCAAAAGTGCCTGTCTT
*Ccl5*(h)	CCAGCAGTCGTCTTTGTCAC	CTCTGGGTTGGCACACACTT
*Cxcl10*(h)	GTGGCATTCAAGGAGTACCTC	TGATGGCCTTCGATTCTGGATT
*Mcp‐1*(h)	CAGCCAGATGCAATCAATGCC	TGGAATCCTGAACCCACTTCT
*Isg15*(h)	GGTGTCCGTGACTAACTCCAT	TGGAAAGGGTAAGACCGTCCT
*Gapdh*(h)	AGGTCGGTGTGAACGGATTTG	TGTAGACCATGTAGTTGAGGTCA
*Isg15*(r)	TTATGAGGCAGGTGTCCCAG	GCTCTGGATAGGGGCCTTAG
*Gapdh*(r)	GGCTCATGACCACAGTCCAT	ACATTGGGGGTAGGAACACG

### 2.4 Western Blot

Following the administration of isoflurane anaesthesia to mice, a systemic perfusion with 20 mL of physiological saline was performed. The heart and aortic tissue were then harvested using ophthalmic scissors. The removal of the fibrous connective tissue surrounding the aorta was then necessary. The aorta was then removed and placed in a tissue grinding dish. Subsequently, the addition of liquid nitrogen was required, followed by grinding. Subsequent to the complete evaporation of the liquid nitrogen, the homogenised dry powder of the aorta was transferred to a 1.5 mL Eppendorf tube. The pre‐chilled RIPA buffer (300 μL RIPA per aorta) was added, followed by sonication on ice for 5 min. The mixture was then left to stand for 30 min. Following this, it was subjected to centrifugation at 12,000 r/min for 15 min. The resultant pellet was collected, and the protein quantified. The protein was added to the 5 × loading buffer (Beyotime, Shanghai) and denatured at 100°C for 5 min. The protein concentration was determined, and the protein sampling, electrophoresis and membrane transfer were then carried out. Following a 30‐min closure period, the relevant primary antibody was introduced and incubated at 4°C on a shaking bed for a duration of 16 h. Thereafter, the secondary antibody was added and incubated at 37°C on a shaking bed for 1 h. Subsequently, ECL chemiluminescence reagent (Beyotime, Shanghai) was incorporated, and images were captured for development. The resulting images were then analysed using ImageJ software. The relative protein expression was determined by utilising the grey value of the target protein bands and β‐actin. Primary antibody information: TRPV1 (1:1000, Sigma, USA), ISG15 (1:1000, Abcam, USA), p16 (1:1000, CST, USA), p21 (1:1000, CST, USA), γH2A.X (1:1000, Sigma, USA), p53 (1:1000, CST, USA), p‐p53 (1:1000, CST, USA), p‐p21 (1:1000, CST, USA), p‐Rb (1:1000, CST, USA), β‐actin (1:5000, CST, USA). Secondary antibody information: Goat Anti‐Mouse IgG, Light‐Chain Specific Antibody (HRP Conjugate) (1:2000, CST, USA), Goat Anti‐Rabbit IgG, Light‐Chain Specific Antibody (HRP Conjugate) (1:2000, CST, USA).

### 2.5 Immunofluorescence Double Labeling

Paraffin sections were dewaxed and hydrated with a gradient ethanol solution (Sinopharm, Shanghai). Following antigen repair, a sealing solution (Beyotime, Shanghai) was added dropwise to the sectioned tissues. The sections were then closed for 30 min at room temperature. The primary antibody mixture was prepared by dropwise addition of CD31 (1:200, PhD, Wuhan) and TRPV1 (1:200, Sigma, USA)/ISG15 (1:100, Abcam, USA)/p16 (1:100, Santa Cruz, USA), or a primary antibody mixture of α‐SMA (1:200, Boster, Wuhan) and TRPV1/ISG15/p16, incubated at 4°C overnight. Following an initial 10‐min soak in PBS (Boster, Wuhan) and three subsequent washes, the relevant fluorescent secondary antibody (Goat anti‐Rabbit IgG (H + L) 488 or Goat anti‐Mouse IgG (H + L) 546) (1:500, Thermo Fisher Scientific, USA) was added and left to incubate at 37°C for a period of 1 h. Finally, the slices were sealed with DAPI‐containing sealer (Beyotime, Shanghai), and images were acquired under a fluorescence microscope to observe TRPV1, ISG15, and P16 staining in vascular endothelial cells and smooth muscle cells.

### 2.6 Senescent Associated Beta‐Gal Staining

The presence of senescent cells in the aortic sinus was detected using the senescence associated β‐galactosidase Staining Kit (SA‐β‐gal Kit) (Beyotime, Shanghai). Frozen sections of aortic sinus tissue were placed in phosphate‐buffered saline (PBS) and washed thrice. Thereafter, the β‐galactosidase staining working solution was added dropwise, and the slices were placed in a wet box and incubated at 37°C for 6 h. The staining was then observed under a microscope. Following this, the slices were soaked in PBS for 5 min and washed thrice. Thereafter, the slices were sealed with a sealing agent (Beyotime, Shanghai), and the pictures were collected under a microscope. The senescent cells exhibited a blue pigmentation.

### 2.7 Elastic Van Gieson (EVG) Staining

Following deparaffinisation of the aortic sinus paraffin sections using xylene (National Pharmaceuticals, Shanghai), gradient ethanol hydration, and sodium citrate antigen repair (Beyotime, Shanghai), the sections were stained with freshly prepared Verhoeff's stain (containing haematoxylin, ferric trichloride, and iodine solution) for 1–3 min at room temperature until the sections were dark black. Following this, the samples were rinsed with distilled water. Then, differentiation was performed with a 2% ferric chloride solution for a period of 10–20 s. The degree of differentiation was then controlled under the microscope until the elastic fibres were black with a light grey background. Following a preliminary wash in distilled water, the reaction was re‐stained with drops of Van Gieson's stain (a mixture of picric acid and acidic magenta) for a period of 10–15 s. The reaction was then rapidly terminated by rinsing with anhydrous ethanol. The samples were then dehydrated quickly and sequentially through anhydrous ethanol (I, II) for 10–15 s per step. Xylene was left to stand for a period of 5 min, after which the neutral gum was sealed. The images were obtained using a light microscope. The ratio of elastic fibres and collagen to the total area of the lumen of the aortic sinus, respectively, was calculated using Image Pro Plus software for the purpose of comparison between groups.

### 2.8 CCK8 Detection of Cell Proliferation

EA.hy926 cells were inoculated in 96‐well plates at a density of 30,000 cells per well. A quantity of 100 μL of cell suspension was added to each well of a 96‐well plate. The plate was set up as follows: a blank group, a control group, and an experimental group, with 3–6 replicate wells in each group. The cells were placed within a 37°C, 5% CO₂ incubator for a period of 24 h. Subsequently, 10 μL of CCK‐8 solution (Beyotime, Shanghai) was added to each well, gently mixed, and the incubation was continued for a further 2 h. The absorbance at 450 nm was measured with an enzyme meter, and the cell proliferation rate was calculated based on the aforementioned measurement.

### 2.9 Angiogenesis Experiment

Pre‐cooled Matrigel (50 μL/well) (BD, USA) was distributed uniformly in a 96‐well plate (operated on ice to prevent solidification) and incubated at 37°C for 60 min to solidify into a gel. The endothelial cells were digested and resuspended, and the density was adjusted to 2 × 10^4^ cells/well. 100 μL of cell suspension was added to each well, and the cells were cultured for 24 h at 37°C in a 5% CO_2_ incubator. The process of vascular lumen formation was observed under the microscope, and 10 photographs were taken for each hole. The number of tubular network nodes was recorded for the purpose of comparison between groups.

### 2.10 Statistical Analysis

ImageJ was utilised for the purpose of image analysis, while SPSS 25.0 was employed for the statistical analysis of the data. Each experiment was repeated independently on more than three occasions, and the measurement data were expressed as x ± s. Comparisons between multiple groups were performed by one‐way ANOVA, and two‐by‐two comparisons between groups were performed by the LSD *t*‐test. A *p* < 0.05 indicated that the difference was statistically significant.

## 3 Results

### 3.1 Reduced TRPV1 Expression in Atherosclerotic Mice

**FIGURE 1:**
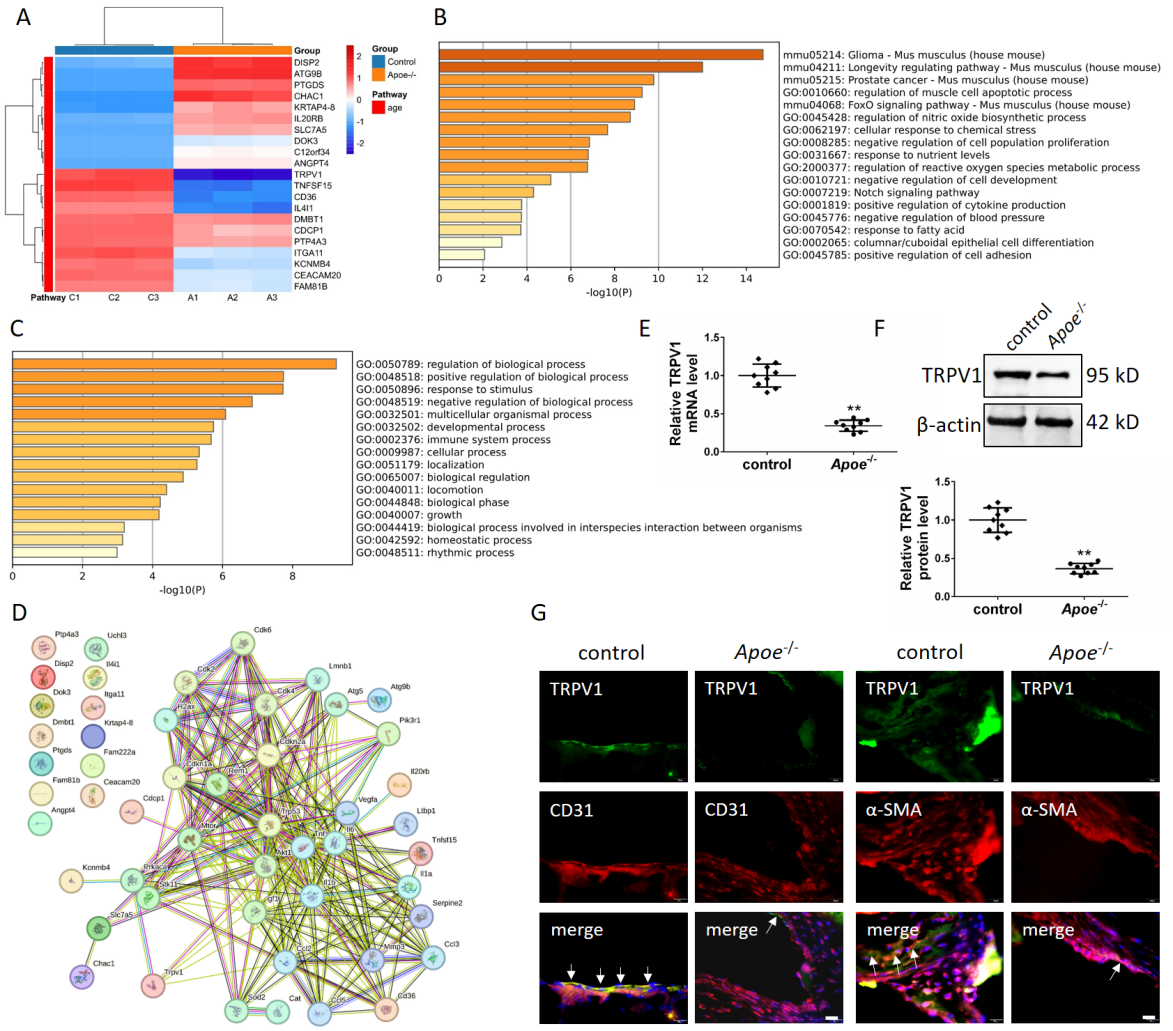
Screening and validation of DEGs in C57BL/6 and *Apoe* ^−/−^ mice. (A) Heat map showing the 21 most DEGs in the aorta between the control and *Apoe* ^−/−^ groups. (B, C) KEGG and GO analysis of DEGs. (D) PPI analysis of the 21 DEGs in relation to genes associated with AS and aging. (E) Real‐time PCR detection revealed that TRPV1 expression in the aortas of *Apoe* ^−/−^ mice on a high‐fat diet was significantly downregulated at the mRNA level. (F) Western blot detection revealed that TRPV1 expression in the aortas of *Apoe* ^−/−^ mice on a high‐fat diet was significantly downregulated. (G) Immunofluorescence double‐labelling detection revealed that TRPV1 expression was significantly reduced in vascular endothelial and smooth muscle cells in the aortic sinus of *Apoe* ^−/−^mice on a high‐fat diet. C57BL/6 mice fed a normal diet were used as the control group and *Apoe* ^−/−^ mice fed a high‐fat diet were used as the *Apoe* ^−/−^ group (AS mice). **p < 0.01. Scale bar: 20 μm.

The present study investigates the differential expression of genes (DEGs) in the aortic tissues of both normal C57 mice and AS model mice through the process of transcriptome sequencing. A total of 258 DEGs were identified. As demonstrated in Figure 1A, a comprehensive list was compiled of the top 10 most significantly upregulated genes and the top 11 most significantly downregulated genes in the model group when compared to the control group. The KEGG and GO analysis of the DEGs between the two groups revealed that the screened DEGs were involved in the regulation of numerous biological functions (Figure 1B,C). Protein interaction (PPI) analysis of the 21 DEGs in Figure 1A (confidence scores of ≥ 0.7, Node Degree: Top 5–10) with AS‐related genes and aging‐related genes revealed that the downregulated DEG *Trpv1* was involved in the regulation of AS and vascular aging (Figure 1D). The present study therefore sought firstly to clarify the differences in the expression of TRPV1 in the aortic tissues of normal C57 mice and AS model mice. The assay revealed that the expression of TRPV1 was significantly lower than that of the control group in the aorta of the model mice, both at the mRNA level (28.33% of the control group) and at the protein level (32.87% of the control group) (Figure 1E,F). These results were in agreement with the results of the transcriptome sequencing. The detection of TRPV1 expression in aortic vascular endothelial cells and smooth muscle cells using immunofluorescence double labeling demonstrated that TRPV1 expression was significantly lower in both model groups than in the control group (Figure 1G). The results of the present study suggest that the downregulation of TRPV1 expression in *Apoe* ^−/−^ mice may be involved in the formation and progression of AS.

### 3.2 TRPV1 Deficiency Promotes Aortic Senescence in AS Mice

**FIGURE 2:**
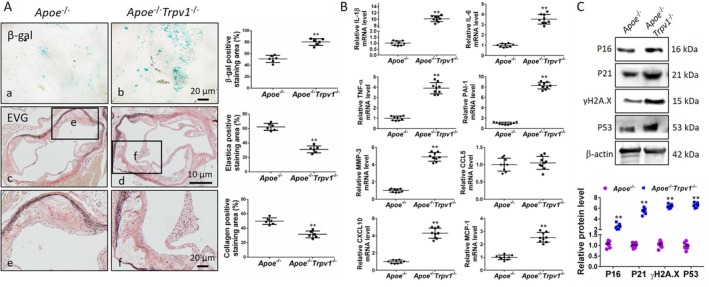
TRPV1 deficiency promotes aortic aging in AS mice. (A) β‐gal staining of the aortic sinus showed that the area of β‐gal positive staining significantly increased after TRPV1 deletion. EVG staining also showed thinner elastic fibres and reduced collagen fibres. (B) In *Apoe* ^−/−^ mice on a high‐fat diet with TRPV1 deletion, the expression of all SASP proteins except CCL5 was significantly increased in aortic tissue. (C) Expression of aging‐related proteins was significantly increased in TRPV1‐deficient AS mice. *p < 0.05, **p < 0.01. Scale bar: 10 μm or 20 μm.

The process of vascular endothelial and smooth muscle cell senescence represents a fundamental catalyst in the development and progression of atherosclerosis. The process of arterial wall dysfunction is driven by a combination of complex cellular autonomous and non‐autonomous mechanisms, working in concert from the earliest stages to advanced plaque formation. Research findings have indicated that hemodynamic dysregulation activates the STING pathway, inducing inflammation and senescence in vascular endothelial cells [29]. This, in turn, results in the disruption of the endothelial barrier and the accumulation of lipids. Concurrently, the decline in nitric oxide (NO) bioavailability caused by endothelial senescence promotes the expression of adhesion molecules on endothelial cells, recruiting monocytes—a key initiating step in AS [30]. Vascular smooth muscle cells undergo metabolic and epigenetic reprogramming with the decline of endogenous protective mechanisms, leading to loss of contractile function. This transformation results in a pro‐inflammatory and pro‐calcification cellular phenotype, which secretes matrix‐degrading enzymes, weakens the fibrous cap, and simultaneously promotes vascular stiffening and remodelling [31, 32]. Subsequently, the effects of downregulation of TRPV1 expression on aortic vascular senescence in AS mice and on pathological structural changes in the vessel wall were observed. SA‐β‐gal staining revealed that the knockdown of TRPV1 led to a significant increase in the stained area of aortic sinus senescence in AS mice compared with *Apoe* ^−/−^ mice. This finding suggests that TRPV1 plays a role in promoting aortic tissue aging. The use of EVG staining to visualise elastic and collagen fibres in tissues revealed a significant reduction in the presence of elastic fibres located in the aortic epicardium in *Apoe* ^−/−^ *Trpv1* ^−/−^ mice, as well as a substantial decrease in collagen fibres located in the aortic mesenchyme. Furthermore, TRPV1 deficiency has been demonstrated to result in increased cholesterol crystallisation within the plaques of AS mice, leading to augmented aortic mesangial vacuoles and heightened plaque instability, which, in turn, has been shown to increase the risk of AS complications (Figure 2A). It was hypothesised that TRPV1 deficiency may exacerbate the progression of AS by promoting vascular tissue senescence. Consequently, the SASP of aortic tissues was examined, as well as changes in the expression of senescence‐associated proteins. The results demonstrated that TRPV1 deletion significantly promoted the expression of pro‐inflammatory cytokines [IL‐1β (increased by 9.20 times), IL‐6 (increased by 3.31 times), TNF‐α (increased by 3.86 times)], adhesion molecules [PAI‐1 (increased by 8.18 times), MMP3 (increased by 4.93 times), CXCL10 (increased by 4.12 times)], and monocyte chemoattractant protein [MCP‐1 (increased by 2.46 times)] at the mRNA level (Figure 2B), as well as the expression of senescence‐associated proteins [p16 (increased by 2.57 times), p21 (increased by 5.16 times), γH2A.X (increased by 6.09 times), and p53 (increased by 6.22 times)] compared with that in the AS model group (Figure 2C). These results suggest that TRPV1 deficiency promotes aortic vascular aging in AS mice while exacerbating AS plaque instability.

### 3.3 RNA‐Sequence Screening of TRPV1 Target Genes Affecting Vascular Aging

**FIGURE 3:**
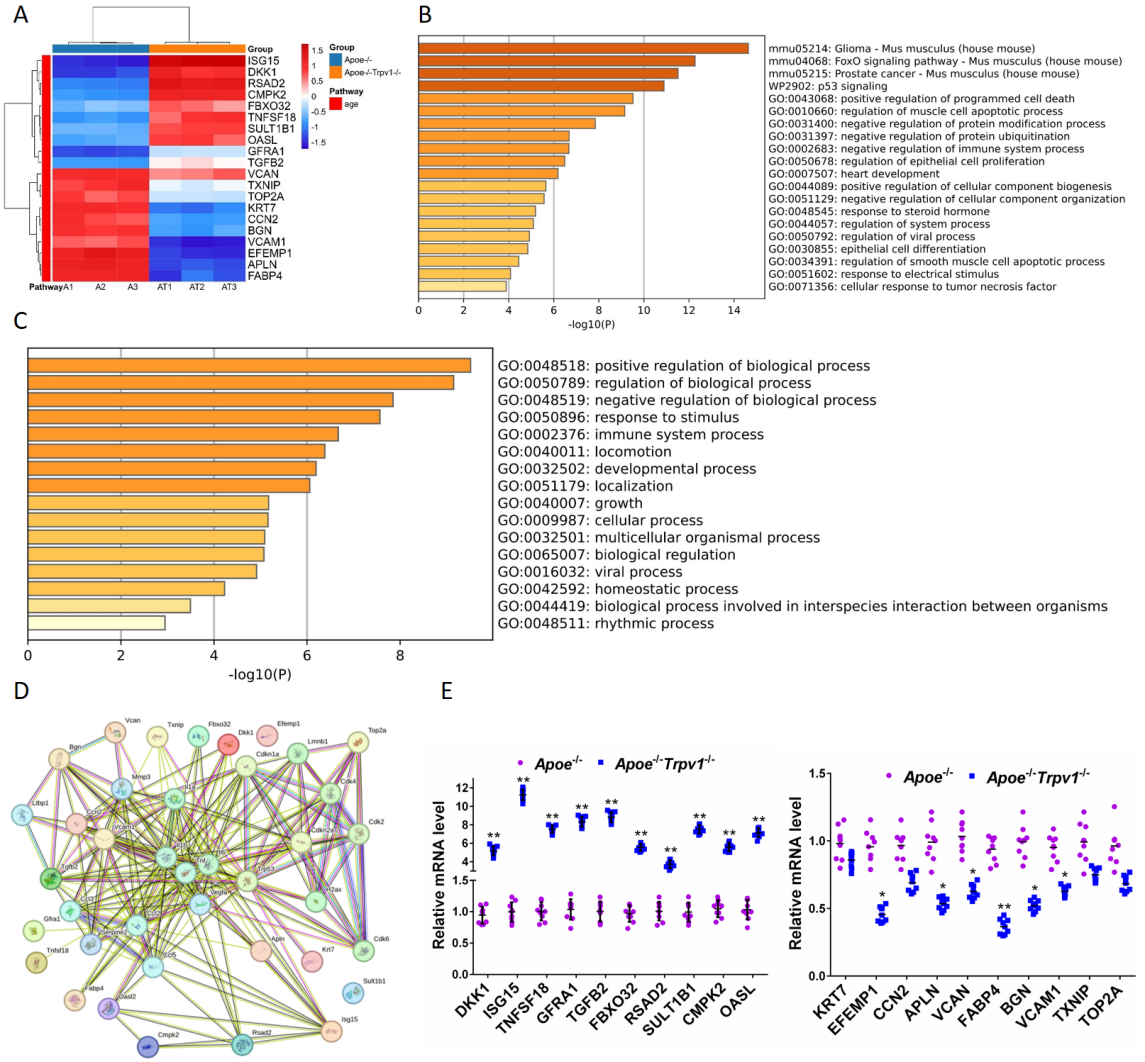
RNA sequencing screening of target genes affected by TRPV1 in vascular aging. (A) Volcano plot showing the top 20 DEGs in AS (*Apoe* ^−/−^ high‐fat diet) and TRPV1‐deficient AS (*Apoe* ^−/−^ *Trpv1* ^−/−^) mice on a high‐fat diet. (B, C) KEGG and GO analysis of the DEGs. (D) PPI analysis of the 20 DEGs and genes related to AS and aging. (E) Real‐time PCR validation of the expression of the 20 DEGs at the mRNA level, with results consistent with RNA sequencing. *p < 0.05, **p < 0.01.

In order to gain insight into which downstream target proteins TRPV1 plays a role in influencing vascular aging in AS mice, we performed transcriptome sequencing of aortic tissues from *Apoe* ^−/−^ and *Apoe* ^−/−^ *Trpv1* ^−/−^ high‐fat‐diet mice and screened for DEGs between the two groups (Figure 3A, up‐regulation and down‐regulation of the top 10 genes according to differential expression ploidy). KEGG analysis (Figure 3B) and GO analysis (Figure 3C) of the aforementioned DEGs, in conjunction with PPI analysis of the DEGs with senescence‐regulating proteins, revealed that ISG15, which was found to be upregulated in the aforementioned DEGs, was involved in the regulation of vascular senescence (Figure 3D). Concurrently, the validation of the aforementioned 20 DEGs was conducted through Real‐time PCR, a method that yielded results consistent with the findings from the transcriptome sequencing. Furthermore, it was observed that the expression of ISG15 exhibited a marked increase (increased by 10.24 times) in *Apoe* ^−/−^ *Trpv1* ^−/−^ mouse aortic tissues, a finding that placed it at the top among all DEGs (Figure 3E).

### 3.4 TRPV1 Deficiency Leads to Increased High‐Fat‐Induced ISG15 Expression

**FIGURE 4:**
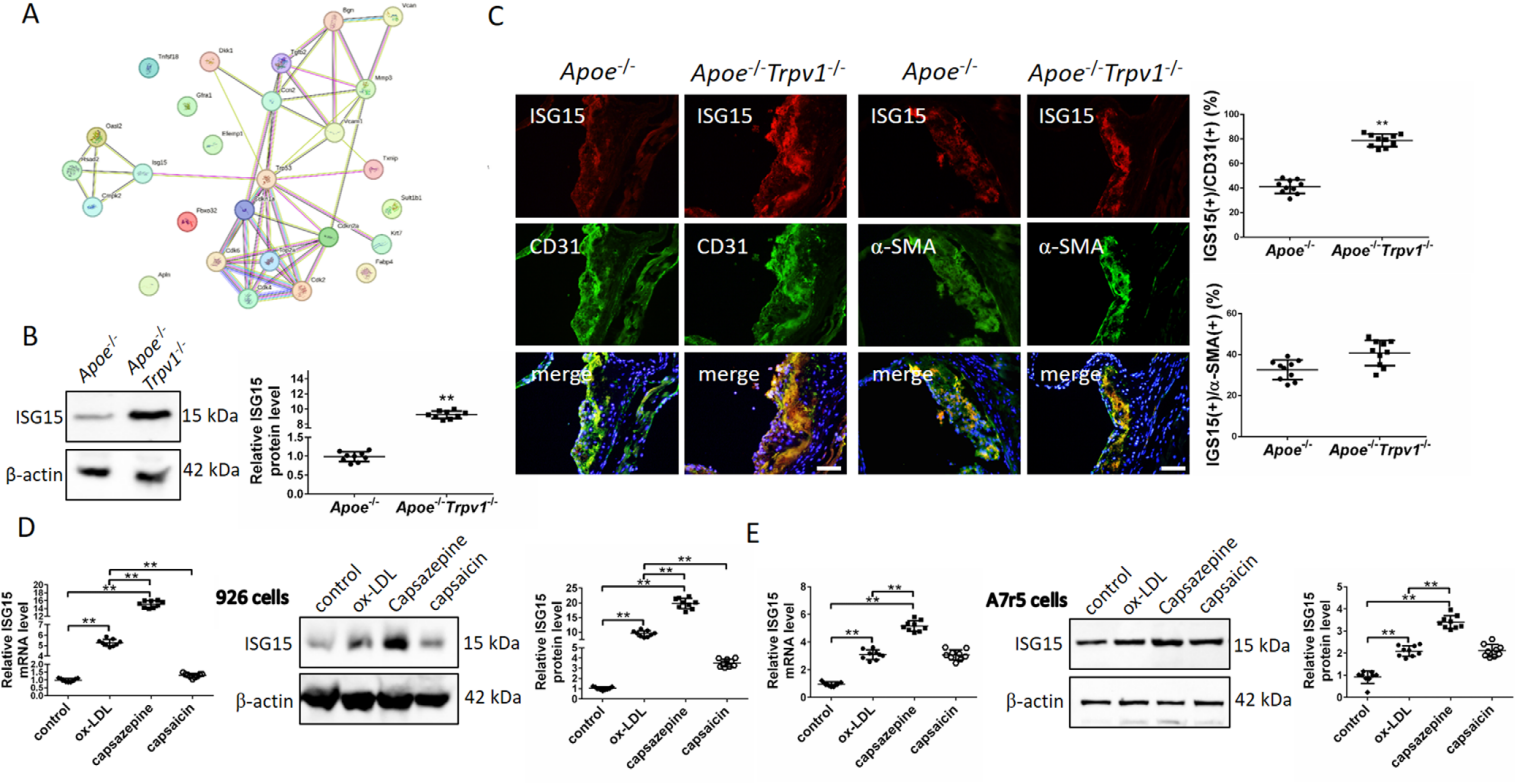
TRPV1 deficiency leads to increased ISG15 expression in the aorta in response to a high‐fat diet. (A) PPI analysis reveals the regulatory relationship between ISG15 and validated DEGs associated with aging‐related proteins. (B) Western blot analysis shows that ISG15 expression in the aortas of *Apoe* ^−/−^ mice fed a high‐fat diet is significantly increased following TRPV1 deficiency. (C) Immunofluorescence double labelling demonstrates that TRPV1 deficiency significantly increases ISG15 expression in the aortic endothelial cells of AS mice. (D) In vitro culture of EA.hy926 cells with the TRPV1 antagonist capsazepine and the TRPV1 agonist capsaicin revealed that capsazepine significantly promoted ISG15 expression, while capsaicin significantly inhibited it. (E) In vitro culture of A7r5 cells with the TRPV1 antagonist capsazepine and the TRPV1 agonist capsaicin showed that capsazepine significantly promoted ISG15 expression, whereas capsaicin failed to inhibit it. **p < 0.01. Scale bar: 50 μm.

In light of the findings from the transcriptome sequencing analysis, a PPI correlation analysis was conducted on ISG15 and senescence regulatory proteins. This analysis revealed that ISG15 plays a role in regulating senescence through the p53 pathway (Figure 4A). The present study hypothesises that TRPV1 deletion exerts an effect on vascular aging by modulating ISG15 expression and, consequently, the p53 signalling pathway. An examination of ISG15 expression in aortic tissues of *Apoe* ^−/−^ and *Apoe* ^−/−^ *Trpv1* ^−/−^ high‐fat diet mice was conducted, resulting in the identification of a significant promotion of aortic ISG15 expression (increased by 9.18 times) in AS mice upon TRPV1 deletion (Figure 4B). Immunofluorescence double labelling was used to characterise the cytological localization and expression of ISG15 in vascular endothelial cells and vascular smooth muscle cells. This revealed that ISG15 was expressed in both vascular endothelial and smooth muscle cells, whereas its expression was significantly increased in TRPV1‐deficient vascular endothelial cells. No difference in expression was observed between the two groups in vascular smooth muscle cells (Figure 4C). The composition of aortic tissue is primarily constituted by two distinct cell types, each exhibiting a unique function. These cell types include vascular endothelial cells and smooth muscle cells. In order to elucidate the effect of TRPV1 on ISG15 expression in these two cell types, this study employed an ox‐LDL model to simulate in vivo lipid injury in atherosclerotic arteries. In order to validate the immunofluorescence double‐staining results, following ox‐LDL‐induced cellular injury, TRPV1 antagonists (capsazepine) and agonists (capsaicin) were administered separately in order to observe their independent effects on ISG15 expression in the aforementioned cell types (EA.hy926 and A7r5 cells). The results of the study demonstrated that capsazepine significantly promoted the expression of ISG15 in both EA.hy926 (increased by 20.12 times at protein level) and A7r5 cells (increased by 3.35 times at protein level). In contrast to the model group, capsaicin significantly inhibited ISG15 expression only in EA.hy926 cells (decreased by 2.69 times at protein level) (Figure 4D,E). In light of the aforementioned findings, a hypothesis was formulated proposing that ISG15 may assume a more pivotal function in the regulation of vascular endothelial senescence. Consequently, an in‐depth investigation into the mechanism of the TRPV1‐ISG15 pathway in regulating vascular endothelial cell senescence is warranted.

### 3.5 ISG15 Is Involved in the Regulation of Vascular Endothelial Cell Senescence

**FIGURE 5:**
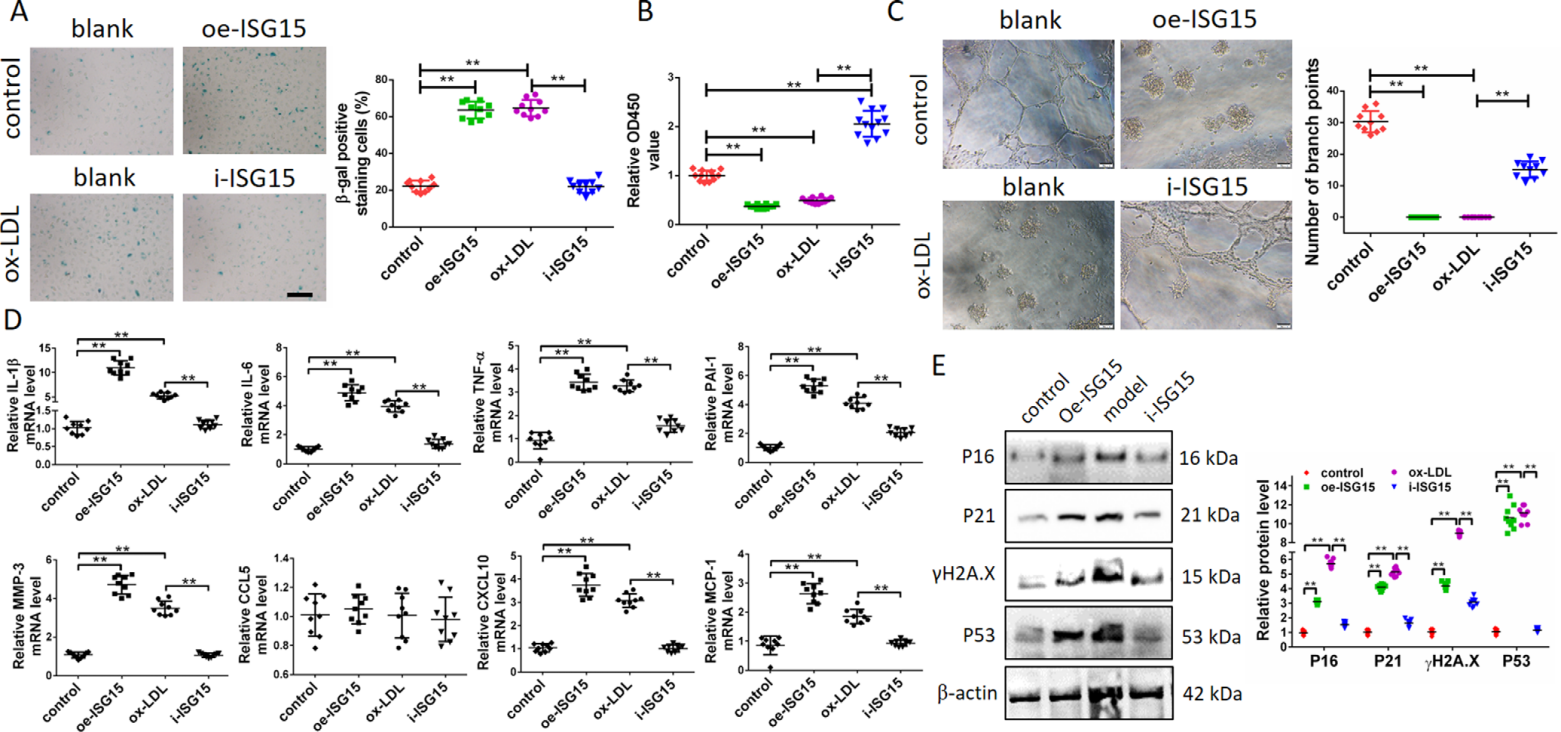
ISG15 participates in the regulation of vascular endothelial cell senescence. (A) β‐gal staining shows that ISG15 overexpression promotes senescence in normal cells, while ISG15 interference in damaged cells inhibits senescence. (B) A CCK8 assay shows that ISG15 overexpression inhibits cell proliferation, while ISG15 interference in damaged cells promotes cell proliferation. (C) The angiogenesis assay shows that ISG15 overexpression inhibits angiogenesis, while ISG15 interference in damaged cells promotes angiogenesis. (D) Real‐time PCR detection of SASP secretion showed that ISG15 overexpression promoted SASP secretion, while ISG15 interference in damaged cells inhibited SASP secretion. (E) In vascular endothelial cells, ISG15 overexpression promoted the secretion of age‐related proteins, while ISG15 interference in damaged cells inhibited the secretion of age‐related proteins. *p < 0.05, **p < 0.01. Scale bar: 50 or 100 μm.

The over‐expression of ISG15 in normal cultured EA.hy926 cells (control group) was recorded as the oe‐ISG15 group, and the interference with ISG15 in EA.hy926 cells with ox‐LDL added (model group) was recorded as the i‐ISG15 group. Intergroup comparisons revealed significant variations in cellular senescence, proliferation, and angiogenesis, as well as the expression of SASP and senescence‐associated proteins. The results demonstrated that the over‐expression of ISG15 significantly promoted cellular senescence (increased by 41.3%), and interference with ISG15 in senescent cells resulted in a substantial suppression of cellular senescence (decreased by 42.7%) (Figure 5A), and concomitantly inhibited cell proliferation (Figure 5B) and angiogenesis (Figure 5C) in EA.hy926 cells compared to the control group. The expression of SASPs [IL‐1β (increased by 10.55 times), IL‐6 (increased by 4.68 times), TNF‐α (increased by 3.39 times), PAI‐1 (increased by 5.26 times), MMP3 (increased by 4.59 times), CXCL10 (increased by 3.61 times) and MCP‐1 (increased by 2.63 times)] (Figure 5D) and senescence‐related proteins [p16 (increased by 3.15 times), p21 (increased by 4.14 times), γH2A.X (increased by 4.28 times) and p53 (increased by 10.36 times)] (Figure 5E) were also promoted compared to the control group. In contrast, the ability of ox‐LDL to promote senescence, inhibit proliferation and angiogenesis, and promote the expression of SASP [IL‐1β (decreased by 86.08%), IL‐6 (decreased by 73.69%), TNF‐α (decreased by 64.30%), PAI‐1 (decreased by 46.83%), MMP3 (decreased by 95.02%), CXCL10 (decreased by 125.56%) and MCP‐1 (decreased by 107.31%)] and senescence‐associated proteins [p16 (decreased by 62.97%), p21 (decreased by 59.17%), γH2A.X (decreased by 33.85%) and p53 (decreased by 86.23%)] was significantly attenuated in EA.hy926 cells after interfering with ISG15 compared with the model group. The results of the present study indicate that elevated levels of ISG15 expression are associated with the promotion of vascular endothelial cell senescence. However, further research is required to elucidate the precise regulatory mechanisms by which ISG15 promotes cellular senescence.

### 3.6 Inhibition of Aortic Aging by Interfering With ISG15 Expression in TRPV1‐Deficient Mice

**FIGURE 6:**
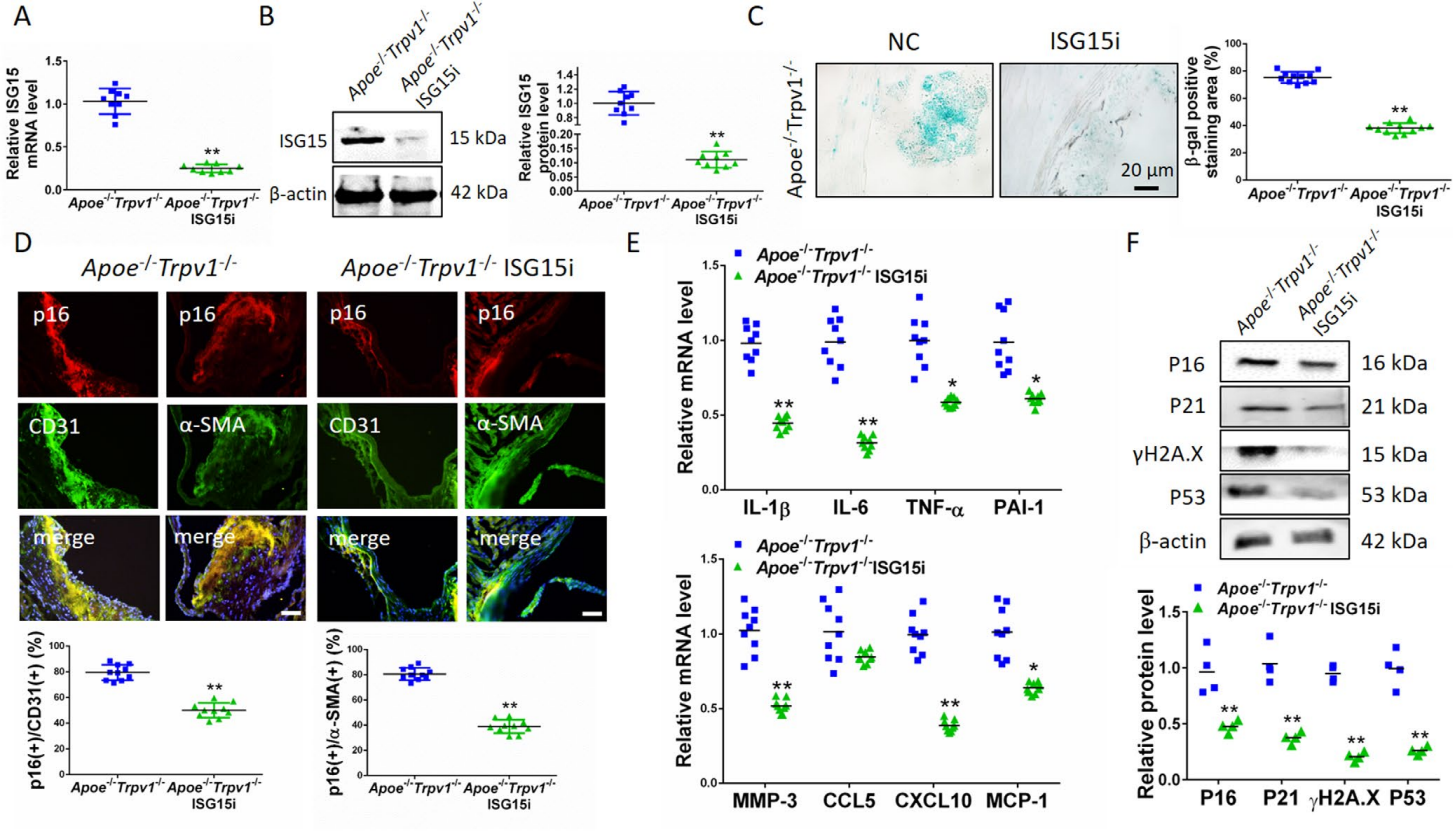
Interference with ISG15 expression inhibits vascular aging in TRPV1‐deficient mice. (A) Real‐time PCR validation of ISG15 expression at the mRNA level in *Apoe* ^−/−^ *Trpv1* ^−/−^ mice, showing significant downregulation of ISG15 expression after interference. (B) Western blot validation of ISG15 expression at the protein level in *Apoe* ^−/−^ *Trpv1* ^−/−^ mice after ISG15 interference, showing significant downregulation of ISG15 expression. (C) β‐gal staining showed that the area of aged cells in the aortic sinus of mice was significantly reduced following ISG15 interference. (D) Immunofluorescence double‐labelling staining showed that p16 expression was significantly downregulated in both vascular endothelial and smooth muscle cells of the aortic sinus after ISG15 interference. (E) Real‐time PCR detection of SASP expression in mouse aortic tissue showed that the expression of all other SASP proteins was significantly downregulated after ISG15 interference, except for CCL5, which remained unchanged. (F) Western blot detection of aging‐related protein expression in mouse aortic tissue showed that, after ISG15 interference, the expression of all aging‐related proteins was significantly reduced. **p < 0.01. Scale bar: 20 or 50 μm.

It has been established through previous studies that TRPV1‐deficient AS mice exhibit heightened ISG15 expression in aortic tissues and accelerated senescence. Consequently, further elucidation is necessary to ascertain whether TRPV1 instigates aortic senescence via ISG15. In the present study, we injected cardiovascular system‐targeted AAV9 viruses containing the ISG15 interference plasmid into *Apoe* ^−/−^ *Trpv1* ^−/−^ mice and observed the expression level of ISG15 is significantly downregulated in *Apoe* ^−/−^ *Trpv1* ^−/−^ mice on a high‐fat diet after interference with ISG15 (Figure 6A,B). The results of the study revealed that, under conditions of a high‐fat diet, interference with ISG15 in *Apoe* ^−/−^ *Trpv1* ^−/−^ mice resulted in a significant reduction in the area of β‐Gal‐positive staining within the aortic sinus, with a decrease of 37.35% compared to levels observed prior to the intervention (Figure 6C). The immunofluorescence double‐label staining method was utilised to observe vascular endothelial and smooth muscle cell senescence. The results demonstrated that following interference with ISG15 in *Apoe* ^−/−^ *Trpv1* ^−/−^ mice, the number of p16‐positive senescent cells was significantly reduced in both vascular endothelial cells (decrease by 30.66%) and vascular smooth muscle cells (decrease by 41.59%) (Figure 6D). The expression of both the SASP—which includes IL‐1β (decreased by 54.23%), IL‐6 (decreased by 68.14%), TNF‐α (decreased by 42.43%), PAI‐1 (decreased by 38.45%), MMP3 (decreased by 47.8%), CXCL10 (decreased by 61.53%) and MCP‐1 (decreased by 36.2%) (Figure 6E) – and the expression of senescence‐associated proteins [p16 (decreased by 67.97%), p21 (decreased by 59.17%), γH2A (decreased by 33.85%) and p53 (decreased by 86.23%)] (Figure 6F) were significantly reduced. The results presented above indicate that interfering with ISG15 expression in TRPV1‐deficient AS mice significantly inhibited aortic aging. This suggests that TRPV1 affects aortic aging in AS mice through ISG15.

### 3.7 TRPV1 Targeting ISG15 Is Involved in the Regulation of Vascular Endothelial Cell Senescence

**FIGURE 7:**
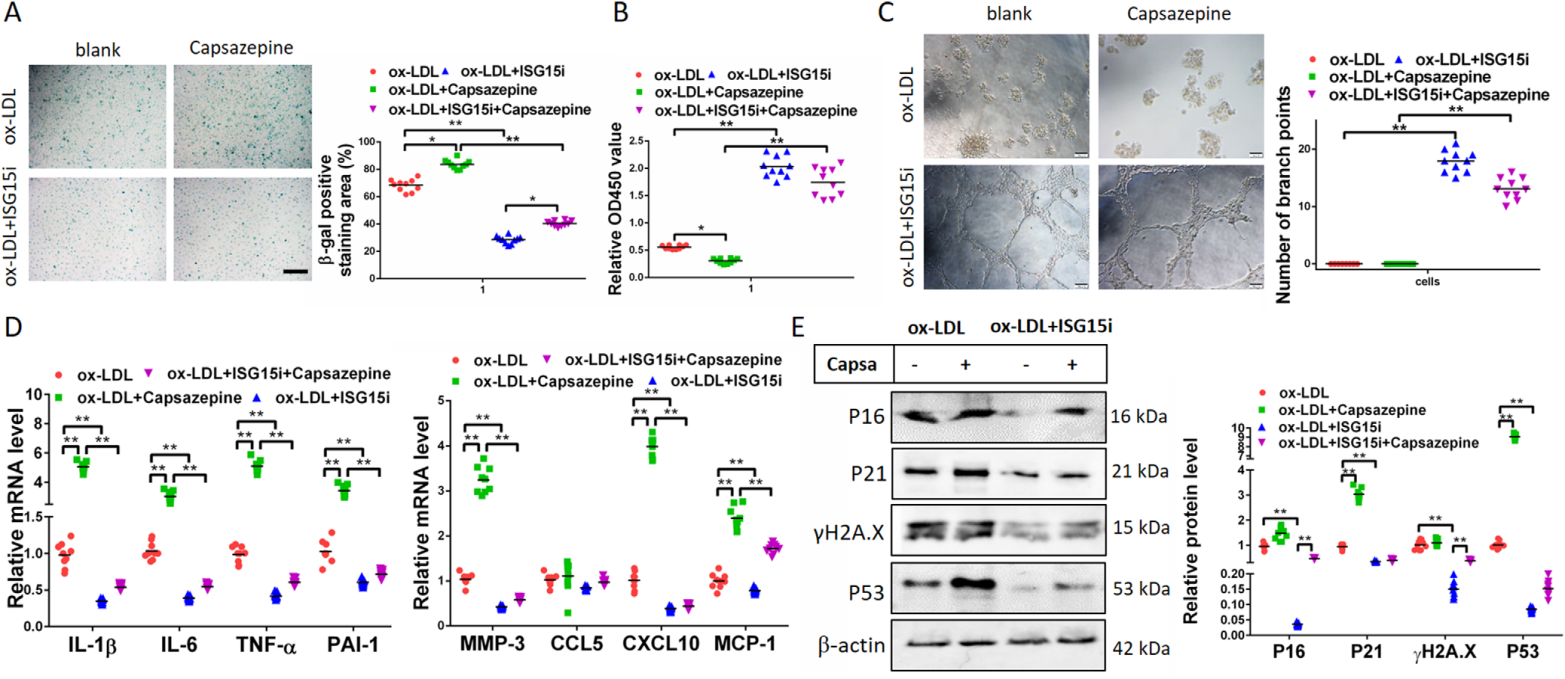
TRPV1 targets ISG15 to regulate vascular endothelial cell senescence. (A) β‐gal staining shows that the TRPV1 antagonist capsazepine exacerbates ox‐LDL‐induced cell senescence. When ISG15 is interfered with, however, the senescence‐promoting effect of capsazepine is significantly weakened. (B) Capsazepine significantly enhances the ox‐LDL‐inhibited cell proliferation effect, while interfering with ISG15 significantly reduces this effect of capsazepine. (C) In EA.hy926 cells with interfered ISG15, the inhibitory effect of capsazepine on angiogenesis was significantly reduced. (D) In EA.hy926 cells with interfered ISG15, the promoting effect of capsazepine on SASP (excluding CCL5) expression was significantly weakened. (E) Capsazepine significantly increased the expression of p21 and p53, but had no effect on the expression of p16 and γH2A.X. After ISG15 was interfered with, capsazepine only increased the expression of p16 and γH2A.X, and had no effect on the expression of p21 and p53. *p < 0.05, **p < 0.01. Scale bar: 50 or 100 μm.

Furthermore, we conducted experiments in EA.hy926 cells cultivated in vitro to ascertain whether hindering ISG15 in the presence of TRPV1 antagonist exerts an effect on cellular senescence. In this study, we employed an in vitro model of cellular injury by adding ox‐LDL to EA.hy926 cells. We then investigated the impact of TRPV1 loss of function on cellular senescence. Our findings revealed that this loss significantly increased the number of senescent cells. Furthermore, we observed a significant reduction in the number of senescent cells when we added ox‐LDL to cells interfering with ISG15, as compared to the model group. At this juncture, capsazepine was reintroduced, and it was ascertained that interference with ISG15 still significantly inhibited cellular senescence (Figure 7A). In a similar manner, the use of CCK8 and angiogenesis assays revealed that capsazepine significantly inhibited cell proliferation and angiogenesis. However, this inhibition was significantly attenuated by interfering with ISG15 (Figure 7B,C). Real‐time PCR experiments to detect SASPs in aortic tissues revealed that, with the exception of CCL5, the remaining SASPs [IL‐1β (increased by 5.22 times), IL‐6 (increased by 2.95 times), TNF‐α (increased by 5.24 times), PAI‐1 (increased by 3.46 times), MMP3 (increased by 3.27 times), CXCL10 (increased by 4.13 times), and MCP‐1 (increased by 2.36 times)] were significantly increased by the addition of capsazepine, whereas they were significantly decreased by interference with ISG15 (Figure 7D). With regard to the detection of vascular endothelial cell senescence‐related proteins, capsazepine significantly promoted the expression of p21 (increased by 3.13 times) and p53 (increased by 9.22 times). Notably, the expression of p53 increased by approximately 10‐fold; however, no effect was observed on the expression of p16 and γH2A.X in comparison with the cell injury model group. The reintroduction of capsazepine into cells has been shown to disrupt the ISG15 pathway, resulting in elevated levels of p16 and H2A.X expression, while leaving p21 and p53 expression unaltered (Figure 7E). This finding suggests that the regulation of cellular senescence in in vitro‐cultured EA.hy926 cells occurs through the TRPV1‐ISG15‐p53‐p21 pathway.

### 3.8 ISG15 Promotes p53 Phosphorylation to Induce Vascular Endothelial Cell Senescence

**FIGURE 8:**
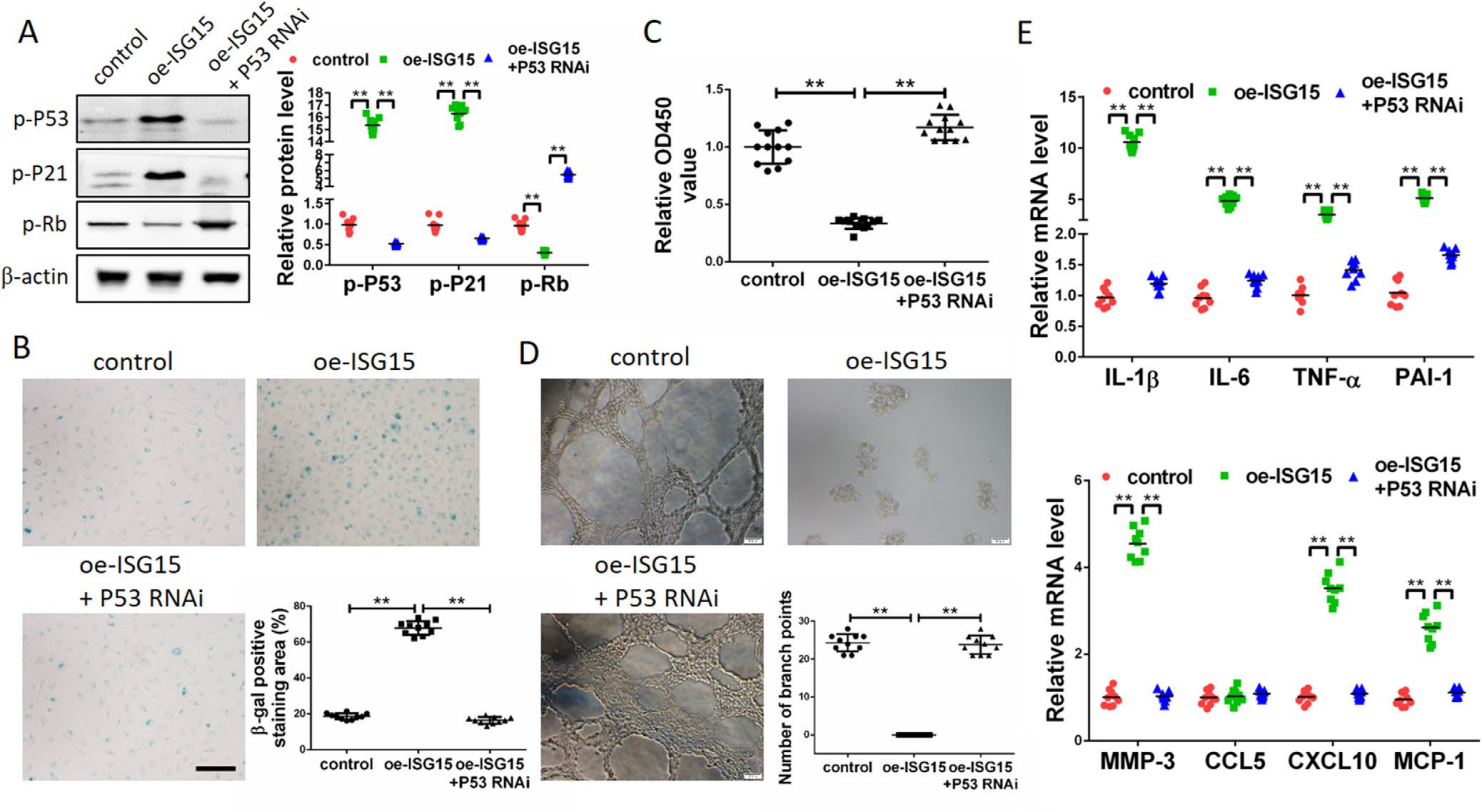
ISG15 promotes vascular endothelial cell senescence by activating p53. (A) Overexpression of ISG15 in EA.hy926 cells promotes the phosphorylation of p53 and p21, while inhibiting the phosphorylation of Rb. After interfering with p53, the phosphorylation of p53 and p21 is weakened, while the phosphorylation of Rb is enhanced. (B) Interfering with p53 significantly inhibited ISG15's promotion of cellular senescence. (C) The inhibitory effect of ISG15 on cell proliferation was significantly weakened after p53 was interfered with. (D) Interfering with p53 significantly inhibited the inhibitory effect of ISG15 on angiogenesis. (E) Interfering with p53 significantly inhibited ISG15's promotion of SASP secretion in vascular endothelial cells. *p < 0.05, **p < 0.01. Scale bar: 50 or 100 μm.

In order to provide further clarification regarding the regulatory mechanism of ISG15 in promoting cellular senescence, an experiment was conducted in which ISG15 was overexpressed in EA.hy926 cells cultured in vitro. The results of this experiment revealed the phosphorylation of p53, p21, and Rb, the latter of which is involved in the cell cycle regulatory protein. The results demonstrated that ISG15 promoted the phosphorylation of p53 (increased by 15.25 times) and p21 (increased by 16.48 times), while concomitantly inhibiting the phosphorylation of Rb (decreased by 69.42%) in EA.hy926 cells. Reduced levels of Rb phosphorylation have been demonstrated to result in cell cycle arrest, which in turn has been shown to promote cellular senescence. In cells that overexpress ISG15, interference with p53 led to a reduction in p53 phosphorylation (decreased by 48.76%). This, in turn, inhibited p21 phosphorylation (decreased by 33.15%) and consequently promoted Rb phosphorylation (increased by 5.68 times). This, in turn, resulted in the entry of the cell cycle into the S‐phase and the reduction of senescent cells (Figure 8A,B). In a similar manner, an investigation was conducted into the effect of the ISG15‐p53 pathway on cell proliferation and angiogenesis. The results of this investigation demonstrated that the overexpression of ISG15 in EA.hy926 cells resulted in a significant inhibition of cell proliferation and angiogenesis, which were found to be markedly enhanced when p53 was interfered with (Figure 8C,D). The present study demonstrates that the overexpression of ISG15 and the interference with p53 have a similar effect on SASP. The overexpression of ISG15 significantly increased the levels of IL‐1β, IL‐6, TNF‐α, PAI‐1, MMP3, CXCL10, and MCP‐1 secreted by EA.hy926 cells, whereas the interference with p53 significantly inhibited the secretion of SASP induced by ISG15 overexpression [IL‐1β (decreased by 88.26%), IL‐6 (decreased by 73.52%), TNF‐α (decreased by 58.23%), PAI‐1 (decreased by 68.38%), MMP3 (decreased by 75.29%), CXCL10 (decreased by 68.07%), and MCP‐1 (decreased by 55.89%)] (Figure 8E). In consideration of the aforementioned findings, it was determined that the deletion of TRPV1 in vascular endothelial cells was conducive to the expression of ISG15. The role of ISG15 in this process is to promote cellular senescence by facilitating the phosphorylation of p53 and p21, while concurrently impeding the phosphorylation of Rb. In aortic tissues of AS mice, TRPV1 deletion led to increased ISG15 production, which in turn contributed to aortic tissue senescence by promoting the p53‐p21 signalling pathway (See the graphical abstract for details.).

## 4 Discussion

Vascular aging is a complex, multifactorial process which occurs alongside the progression of physiological age, exerting a profound influence on the health status of the individual. The incidence of cardiovascular disease has continued to rise in recent years, thus rendering the study of vascular aging a central topic in medicine and biology. A significant number of studies have been conducted to explore the mechanisms of vascular aging and its association with various diseases. For instance, the process of sclerosis of endothelial cells has been demonstrated to play a pivotal role in the development of cardiovascular disease and the progression of vascular aging [33]. As indicated by the extant literature, age‐related changes in vascular aging manifest at the cellular level, with the hallmarks of endothelial dysfunction, cellular senescence, and vascular smooth muscle cell transdifferentiation [34]. A study analysed how vascular aging increases the risk of ischemic stroke in older adults, highlighting the deleterious effects of endothelial dysfunction influenced by oxidative stress and inflammatory responses on vascular aging and ischemic stroke [35]. TRPV1 is traditionally characterised as a nociceptive ion channel, but its role in the vasculature is increasingly being recognised. Our initial observation of reduced TRPV1 expression in the aortas of *Apoe* ^−/−^ mice is consistent with the idea that the loss of protective mechanisms is a feature of vascular disease. Even more compellingly, the accelerated aortic aging, increased secretory senescence‐associated secretory phenotype (SASP) and enhanced plaque instability in *Apoe* ^−/−^ *Trpv1* ^−/−^ mice provide direct genetic evidence that TRPV1 is an active regulator of vascular health, not merely a passive biomarker. Its deficiency creates a permissive environment for accelerated aging, likely by disrupting calcium homeostasis and downstream signalling pathways that maintain cellular integrity. This establishes TRPV1 as a critical sentinel against stress‐induced vascular aging [36].

TRPV1 is a pivotal cation channel that is extensively present in mammalian sensory nerve endings and a variety of tissues. Since the initial discovery of the role of TRPV1 channels in physiological and pathological processes, the field has attracted the interest of a wide range of researchers. It is important to note that this process is not only responsive to conventional stimuli such as temperature, pain, and inflammation, but has also been implicated in the development of numerous diseases, including cardiovascular, neurodegenerative, and digestive diseases [4, 5]. TRPV1 was found to play a key role in regulating vascular tone, promoting angiogenesis, antifibrosis, anti‐inflammation, and antioxidant mechanisms. The regulatory mechanisms of TRPV1 were mainly related to intracellular calcium regulation, nitric oxide, and calcitonin gene‐related peptide release [6]. Consequently, the results of this study augment the existing knowledge concerning the function of TRPV1 in non‐neural systems, thereby establishing it as an endogenous vascular protective factor. Its downregulation has been hypothesised to represent a critical link in the failure of vascular defence mechanisms against stressors, such as high‐fat diets.

ISG15 is an ubiquitin‐like protein that functions by binding to target proteins (ISGylation) or as a free or non‐binding protein [37]. ISGylation is a process that involves a series of enzymatic reactions catalysed by the E1, E2, and E3 enzymes. These enzymes facilitate the binding of ISG15 to lysine residues within the target protein [38]. *Isg15* gene deletion has been demonstrated to result in the accumulation of misfolded/dominant negative p53 and the inhibition of overall p53 activity, consequently leading to a reduction in DNA damage‐induced senescence, an acceleration in cellular proliferation, and a decrease in p21 expression [39]. The investigation revealed that *Isg15* ^−/−^ mice exhibited augmented cell proliferation in vivo, while mouse‐derived cells demonstrated diminished expression of p53 targets and repressed apoptosis [40]. The ISG15‐specific isopeptidase USP18 has been shown to accumulate misfolded p53 through its protease activity, which is required for p53 degradation. Depletion of ISG15 has been observed to result in the accumulation of misfolded dominant‐negative p53 [18]. The precise mechanism through which ISG15 regulates aortic tissue senescence in AS mice remains to be elucidated. The most significant mechanistic insight from this work is the identification of ISG15 as the key downstream effector of TRPV1 in the context of vascular aging. The application of RNA‐sequencing revealed that TRPV1 deficiency specifically and significantly upregulates ISG15 expression in aortic tissue. While ISG15 is best known for its interferon‐induced, ubiquitin‐like role in antiviral immunity, the present study reveals a hitherto unappreciated function in driving cellular senescence within the vasculature. The rescue experiment, in which the interference with ISG15 expression mitigated aortic aging in TRPV1‐deficient mice, unequivocally establishes ISG15 as a central pathogenic driver in this pathway, rather than merely an associated marker. This study also confirmed through in vivo and in vitro experiments that ISG15 acts as an “amplifier” of aging signals in the context of vascular aging.

P53 is a key tumour suppressor protein that plays a central role in regulating cellular senescence [41]. It responds to cellular stress, such as DNA damage, telomere shortening and oncogene activation [42], and determines the fate of the cell (apoptosis, senescence or repair) through multiple mechanisms [43]. P53 is activated in response to DNA damage, oxidative stress and oncogene activation. It then induces cell cycle arrest, apoptosis or senescence by regulating the expression of downstream target genes. As a transcription factor, p53 regulates hundreds of genes, including cell cycle inhibitors (e.g., p21), genes related to apoptosis (e.g., PUMA and BAX), and components of the SASP [44]. It has been demonstrated that p53 activation increases the expression of the cyclin‐dependent kinase inhibitor p21 (CDKN1A). P21 inhibits the activity of CDK2/4/6, resulting in the hypophosphorylation of Rb proteins. This blocks the cell cycle transition from the G1 phase to the S phase, triggering irreversible growth arrest. Therefore, the phosphorylation levels of these proteins can serve as markers of cellular senescence [45, 46]. The findings of this study provide a comprehensive framework for understanding the signalling axis. Specifically, ISG15 has been shown to promote p53 phosphorylation, which in turn leads to the upregulation of the cyclin‐dependent kinase inhibitor p21. Concurrently, it has been demonstrated to contribute to the inhibition of retinoblastoma protein (Rb) phosphorylation. This dual action—activating p53/p21 and inactivating Rb—forcibly arrests the cell cycle, pushing endothelial cells into a persistent senescent state. Senescent endothelial cells exhibit a loss of normal function and secrete a plethora of SASP factors, which in turn promote local inflammation, extracellular matrix degradation, and immune cell recruitment. This process directly explains the increased plaque area and instability observed in the models.

This pathway is of considerable pathophysiological significance. Firstly, it establishes a direct molecular link between vascular aging and AS that extends beyond age‐related factors. Secondly, it elucidates a component of the intrinsic cause of AS plaque instability: SASP secreted by aged endothelial cells, such as IL‐6 and MMPs, has the capacity to disrupt the fibrous cap, recruit additional inflammatory cells, and render plaques susceptible to rupture. The increased plaque instability observed in TRPV1 knockout mice in this study directly reflects the ultimate manifestation of this pathway. The study proposes two intervention strategies. The first of these is ‘upstream activation’, which involves the development of selective TRPV1 agonists to mimic its protective effects. Capsaicin, a naturally occurring TRPV1 agonist, provides supporting evidence through its epidemiological association with cardiovascular benefits when consumed in appropriate amounts [47]. The second approach is ‘downstream inhibition’, which involves the functional blockade of ISG15 or the interference with its interaction with p53/p21. This approach may offer novel insights for intervening in the aging process itself.

Further research is required to elucidate the specific molecular mechanisms by which TRPV1 regulates ISG15 transcription (including whether calcium influx, NF‐κB, and other pathways are involved) and to investigate the role of this axis in different cell types, such as vascular smooth muscle cells and macrophages. Looking ahead, there is still much to be discovered in the field of vascular aging through in‐depth research. Firstly, improving our understanding of the molecular mechanisms and biomarkers of vascular aging could help us to prevent and intervene early. Secondly, the characteristics of vascular aging and intervention strategies are studied in different population groups, such as those categorized by gender and age. Furthermore, exploring new treatments such as medication, lifestyle changes, or other interventions that could slow or reverse vascular aging is an important area for future research. In conclusion, the accelerated aging of society makes the study of vascular aging important for preventing and treating cardiovascular and cerebrovascular diseases.

## Author Contributions

**Fangfang Dou:** writing – review and editing, writing – original draft, project administration, funding acquisition, formal analysis, data curation. **Jishuang Liu:** methodology, investigation, formal analysis, data curation, conceptualization, writing – review and editing. **Jiulin Chen:** writing – review and editing, visualization, software, methodology, formal analysis. **Te Liu:** writing – review and editing, data curation, formal analysis, investigation. **Duo Zhang:** writing – review and editing, writing – original draft, validation, supervision, resources, methodology, investigation, conceptualization. **Yun Gu:** writing – review and editing, supervision, funding acquisition, conceptualization, investigation. **ZhiHua Yu:** supervision, writing – review and editing, conceptualization, funding acquisition, resources.

## Funding

The present study was supported by grants from the Shanghai Municipal Health Commission Clinical Research Special Project (202140199).

## Disclosure

Publisher's Note: All claims expressed in this article are solely those of the authors and do not necessarily represent those of their affiliated organisations, or those of the publisher, the editors and the reviewers. Any product that may be evaluated in this article, or claim that may be made by its manufacturer, is not guaranteed or endorsed by the publisher.

## Ethics Statement

The animal study was reviewed and approved by the Animal Care Committee for the use of laboratory animals at the Shanghai University of Traditional Chinese Medicine.

## Conflicts of Interest

The authors declare no conflicts of interest.

## Data Availability

The original contributions presented in the study are included in this article; further inquiries can be directed to the corresponding author.
